# FBXO31 inhibits the stemness characteristics of CD147 (+) melanoma stem cells

**DOI:** 10.1515/biol-2025-1145

**Published:** 2025-08-08

**Authors:** Jian Guan, Shenglin Wu, Renyi Liang

**Affiliations:** Graduate School, Zhejiang Chinese Medical University, Hangzhou, Zhejiang, 310053, China; Department of Plastic Surgery, Lishui People’s Hospital, Lishui, Zhejiang, 323000, China; Department of Chemotherapy Oncology, Lishui People’s Hospital, Lishui, Zhejiang, 323000, China; Department of Plastic Surgery, Lishui People’s Hospital, No.1188 Liyang Street, Liandu District, Lishui, Zhejiang, 323000, China

**Keywords:** melanoma, FBXO31, CD147, migration, invasion, stemness

## Abstract

Determining the critical elements in melanoma stem cell growth could aid in preventing the development of malignant cancer. The purpose of this study was to illustrate how FBXO31 affects the stemness, invasion, and migratory properties of melanoma stem cells. Side population (SP) cells with tumor stem cell characteristics were sorted from A375 melanoma cells. The mRNA and protein expression levels of FBXO31 in SP cells were detected using molecular techniques. FBXO31 was then transfected into SP cells, and the proportion of CD147 (+) cells in SP cells was detected by flow cytometry. FBXO31 was also transfected into CD147 (+) cells, and their spheroid formation, migration, and invasion ability were measured. Additionally, CD147 (−) and CD147 (+) cells were inoculated into nude mice to assess the effect of FBXO31 on tumor growth and metastasis. The findings demonstrate that FBXO31 is downregulated in SP cells. Upon FBXO31 transfection, the proportion of CD147 (+) cells sorted from SP cells decreased. CD147 (+) cells exhibit higher stemness characteristics, migration, and invasion abilities than CD147 (−) cells. However, these characteristics were markedly suppressed following FBXO31 transfection in CD147 (+) cells. *In vivo* experiment further showed that CD147 (+) cells promoted tumor growth and metastasis, while after transfection with FBXO31, tumor proliferation and metastatic abilities were inhibited. Overall, FBXO31 inhibits the migration, invasion, and stemness characteristics of CD147 (+) melanoma stem cells.

## Graphical abstract

**Figure:**
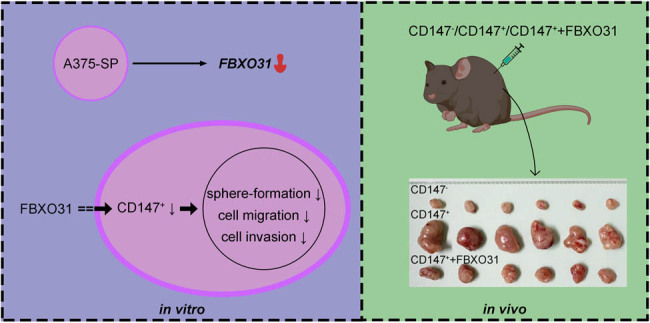

## 1 Introduction

Melanoma is a highly aggressive and metastatic tumor that arises from the malignant transformation of melanocytes [1]. While it frequently affects the skin, it can also affect the mucous membranes. Multiple factors contribute to the development of melanoma, including ultraviolet (UV) exposure, immunosuppression, genetics, environmental influences, and lifestyle factors [2]. Primary treatments for melanoma include tumor surgery, immunotherapy, targeted therapy, radiation, and chemotherapy. Despite these approaches, patients with metastatic melanoma have a poor prognosis. This underscores the urgent need for novel therapeutic interventions.

A small subset of tumor cells within the tumor microenvironment, known as cancer stem cells (CSCs), is distinguished by their capacity for self-renewal, unrestricted replication, and treatment resistance. Evidence suggests CSC’s potent migratory and invasion capabilities as one of the main causes of melanoma metastasis and recurrence [3].

CD147 is a transmembrane protein of the immunoglobulin superfamily. Numerous studies have demonstrated that CD147 is overexpressed in a wide range of cancer types and plays a significant role in the malignant growth of tumors, such as in lung cancer [4], ovarian cancer [5], and breast cancer [6]. In the context of melanoma, CD147 overexpression has been found to encourage metastasis [7] and lymphangiogenesis [8]. A previous study by our team has also demonstrated that CD147 is abundantly expressed in the side population (SP) of melanoma cells and CD147 (+) cells have robust sphere-forming, migration, and invasion capabilities [9].

FBXO31 belongs to the F-box family, which is essential for cancer development. Numerous malignancies exhibit low levels of FBXO31 expression, and FBXO31 can hinder tumor growth. *In vitro*, FBXO31 suppresses the proliferation of cholangiocarcinoma cells and the characteristics of CSCs [10]. Overexpression of FBXO31 has been shown to restrict the development and invasion of glioma cells by accelerating the ubiquitination and degradation of CD147 [11]. In melanoma cells, FBXO31 causes cell cycle arrest and prevents tumor growth *in vivo* [12].

This study confirmed that CD147 (+) cells in melanoma cells have CSC characteristics, and FBXO31 can reduce the CD147 (+) cell subpopulation in SP cells and inhibit the migration, invasion, and stemness characteristics of CD147 (+) cells.

## 2 Methods

### 2.1 Cell culture

A375 cells were cultivated in Dulbecco’s modified eagle’s medium (DMEM) supplemented with 10% fetal bovine serum (FBS), 10,000 units/mL penicillin, and 10,000 μg/mL streptomycin. Cells were maintained in a humidified incubator at 37°C with 5% CO_2_ and passaged every 3–4 days to sustain exponential growth.

### 2.2 Cycloheximide (CHX) chase experiment

Melanoma cells were seeded in six-well plates and cultured to 70–80% confluence. FBXO31 overexpression plasmid was transfected, and the cells were cultured for 24 h following transfection. CHX was dissolved in DMSO and diluted to a final concentration of 50 μg/mL with complete culture medium when used. For CHX treatment, the old culture medium was discarded and fresh culture medium containing CHX was added to each group. Cells were collected at 0, 2, 4, and 8 h, and the CD147 protein was detected by Western blot. The relative expression level at each time point was calculated with 0 h as 1.

### 2.3 Ubiquitination assay

Twenty-four hours post-transfection, MG132 (final concentration 10 μM) was added to the culture medium and incubated for another 6 h. Following treatment, the culture medium was discarded and the cells were washed twice with pre-cooled phosphate-buffered saline (PBS). Lysis buffer was then added and lysed on ice for 30 min. The remaining cells were scraped and centrifuged at 12,000 rpm for 15 min, maintaining 4°C, and the supernatant was collected. Co-immunoprecipitation was used to enrich ubiquitinated CD147, and the ubiquitinated bands were detected by Western blot.

### 2.4 Isolation of SP and non-SP cells

A375 cells were cultured in DMEM until the logarithmic growth phase and then digested with trypsin to prepare a single cell suspension. The cell concentration was adjusted to 1 × 10^6^ cells/mL, and Hoechst 33342 dye was added to a final concentration of 5 µg/mL. The cells were incubated in a 37°C water bath for 90 min, with gentle mixing every 15 min. Staining was terminated by washing the cells twice with pre-cooled PBS and centrifuging at 4°C. The cells were then resuspended in pre-cooled PBS and filtered through a cell screen. SP cells were isolated from melanoma cell populations based on their ability to efflux the DNA-binding dye Hoechst 33342 via ATP-binding cassette transporters. The cells were sorted using a flow cytometer (LSRFortessa, BD Biosciences, USA) based on the Hoechst staining pattern and gated to distinguish between SP cells (low staining) and non-SP cells (high staining).

### 2.5 Cell transfection

Cultivated cells in the logarithmic growth cycle were passaged to six-well plates and cultured for 24 h until the cell density reached 70–80% confluence. Transfection solution was prepared by adding 5 µg of FBXO31 plasmid to a serum-free medium in a sterile tube followed by the addition of Lipofectamine 3000. The tube was mixed well and let stand at room temperature for 15 min. The mixture was then added dropwise to the cell culture wells, and the culture plate was gently shaken to mix evenly. After adding, the transfection mixture culture plate was returned to the CO₂ incubator and incubated for 8 h before replacing the medium with the complete medium. The plate was further incubated for 24 h for complete transfection.

### 2.6 Isolation of CD147 (+) and CD147 (−) cell

SP cells were cultured in complete culture medium until the logarithmic growth phase. Single cell suspension was prepared by resuspending the cells in PBS. Following resuspension, anti-CD147 fluorescent antibody was added to the cells, mixed gently, and incubated for 30 min at 4°C in the dark. Cells were then washed and again resuspended in PBS. CD147 (−) and CD147 (+) cells were sorted using flow cytometry according to the fluorescent signal.

### 2.7 Spheroid formation assay

Cells were seeded into an ultra-low attachment culture plate in stem cell culture medium consisting of DMEM/F12 supplemented with 20 ng/mL epidermal growth factor, 20 ng/mL basic fibroblast growth factor, and 1× B27. The plates were incubated at 37°C with 5% CO_2_. After 10 days of culture, the number of spheres with a diameter greater than 50 µm in each well was counted under a microscope.

### 2.8 Wound healing

Cells were seeded into six-well plates and cultured for 24 h to form a monolayer with 90–100% confluence. A straight line was drawn on the bottom of the culture plate using a marker that acted as a reference line for the wound region. A sterile pipette tip was then used to create a linear scratch (wound) perpendicular to the reference line. Wells were gently washed with PBS to remove detached cells and debris, and serum-free medium was added to each well. A picture of the wound area’s initial condition was taken using an inverted microscope at 0 h. After that, the plates were put back in the incubator maintained at 37°C with 5% CO₂ for additional incubation for 24 h, and the wound healing process was monitored and photographed.

### 2.9 Transwell invasion assay

Matrigel was diluted with serum-free medium, and 50 µL of the diluted solution was evenly applied to the upper surface of the Transwell insert. The inserts were then incubated at 37°C for 1 h to allow the Matrigel to solidify. Complete medium containing 10% FBS was added to the lower chamber, while the upper chamber was loaded with cell suspension. The Transwell plates were incubated at 37°C with 5% CO₂ for 24 h. After incubation, the side of the upper chamber with Matrigel and uninvaded cells were wiped gently with a cotton swab. The inserts were fixed with 4% paraformaldehyde, followed by staining with 0.1% crystal violet solution. The cells in the Transwell’s lower chamber were imaged under an inverted microscope.

### 2.10 PCR

The cells were completely lysed using TRIzol reagent (Invitrogen, 15596026CN), and the RNA was extracted using standard procedure. The extracted RNA was washed and solubilized to measure its concentration using a spectrophotometer. To eliminate genomic DNA contamination, RNA samples were treated with gDNA Eraser. Reverse transcription was subsequently performed using reverse transcriptase and specific primers. The reaction mixture was then prepared in accordance with the SYBR Green qPCR kit’s instructions (Sigma-Aldrich, QR0100). The reactions were run on a real-time quantitative PCR device (QuantStudio 6 Flex, Thermo Fisher Scientific, USA). Relative FBXO31 expression was computed using the 2^−ΔΔCt^ method. The primer sequences for FOXB31 were as follows: forward primer: 5′-AGGCCAGGCTTGATGAGGT-3′ and reverse primer: 5′-ATCTTCCACGAGCACATGCAG-3′.

### 2.11 Western blot

Total protein was extracted from the cell using RIPA lysis buffer (Sigma-Aldrich, 20-188). The protein concentration was determined using a BCA kit (Sigma-Aldrich, 71285-M). After that, the protein was separated using sodium dodecyl sulfate–polyacrylamide gel electrophoresis and transferred to a nitrocellulose membrane. The membrane was blocked with 5% skim milk powder at room temperature and incubated with primary antibodies overnight at 4°C. After washing, the membrane was incubated with horseradish peroxidase-conjugated secondary antibodies and visualized using an enhanced chemiluminescence substrate. The primary antibodies used in the experiment were FBXO31 (Abcam, ab86137, 1:1,000), SOX2 (Abcam, ab92494, 1:1,000), and β-actin (Abcam, ab8227, 1:1,000).

### 2.12 Animals

Nude mice (specific pathogen-free [SPF]-grade, Charles River, China) were acclimated in a SPF animal facility for 1 week prior to experimentation. The mice were then randomly divided into three groups, CD147 (−), CD147 (+), and CD147 (+) + FBXO31, each group containing six mice. Following proper fixation of the mice, cells were subcutaneously injected into the axillary region using a sterile technique. The injection site was disinfected with alcohol prior to administration. Tumor growth was monitored weekly starting from day 7 post-injection. The long (L) and short (W) diameters of the tumors were measured using Vernier calipers, and tumor volume was calculated using the following formula: tumor volume (mm^3^) = (*L* × *W* ^2^)/2. Tumor volume for each naked mouse was measured and used to draw a growth curve. After 35 days, nude mice were euthanized using CO₂. Tumor and lung tissue were harvested, and the lung tissue was stained using hematoxylin and eosin (H&E) stain.


**Ethical approval:** The research related to animal use has been complied with all the relevant national regulations and institutional policies for the care and use of animals, and has been approved by the Ethics Committee of Zhejiang Chinese Medical University (approval no. 2022042).

### 2.13 Statistical analysis

Statistical analysis was performed using SPSS software version 22.0. The quantitative data were compared using the Tukey *post hoc* test and the analysis of variance test, while the *T*-test was employed to compare the two groups.

## 3 Results

### 3.1 FBXO31 is downregulated in melanoma and promotes CD147 ubiquitination

**Figure 1:**
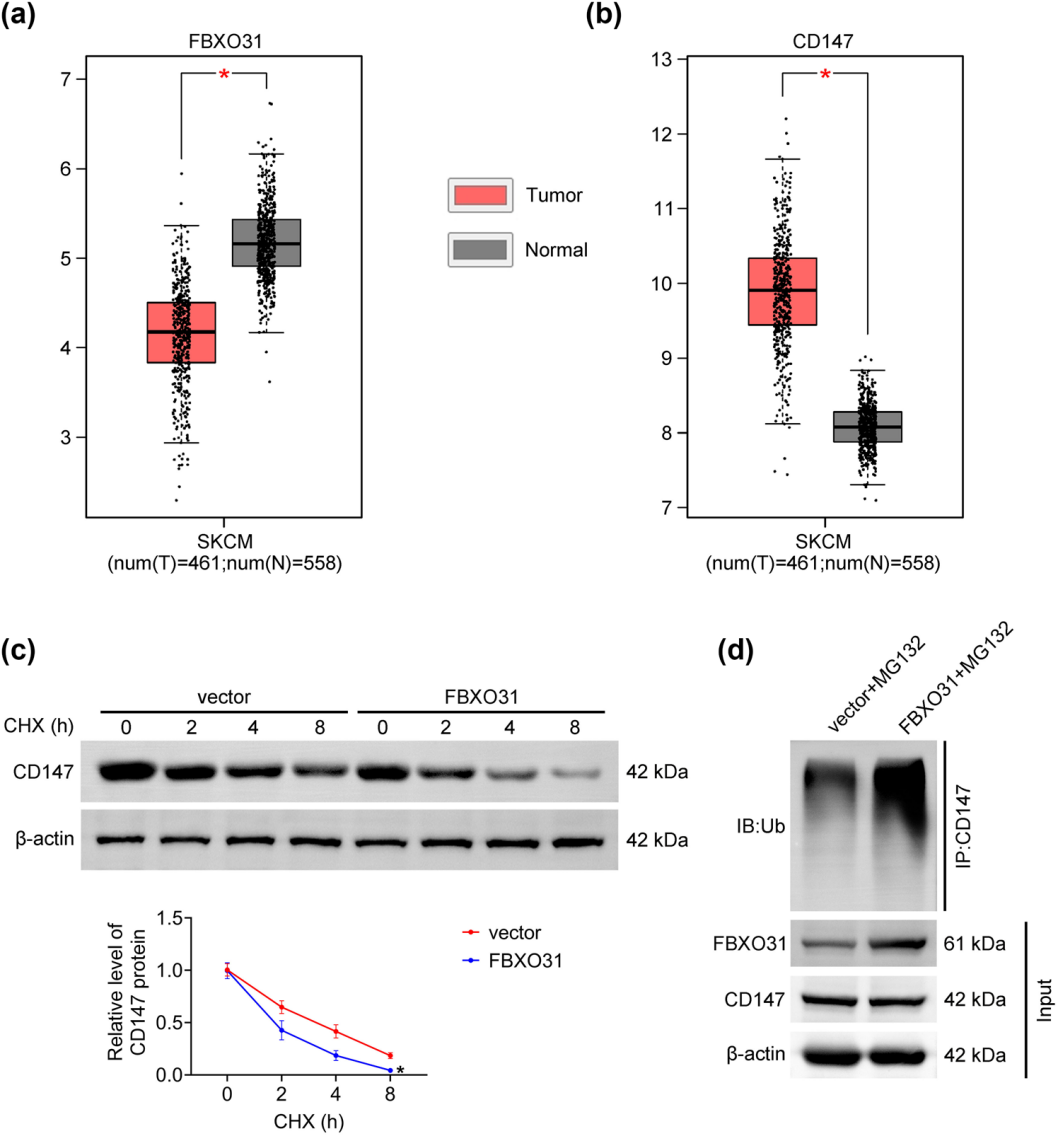
FBXO31 is downregulated in melanoma and promotes CD147 ubiquitination. (a) Expression levels of FBXO31 in skin melanoma tissues and normal tissues in the GEPIA database. (b) Expression levels of CD147 in skin melanoma tissues and normal tissues in the GEPIA database. (c) CHX tracking experiment to detect the effect of transfection of FBXO31 on CD147 protein degradation. (d) Ubiquitination assay to detect the effect of transfection of FBXO31 on CD147 ubiquitination level, vs normal, *P < 0.01; vs vector, *P < 0.05.

Analysis of the TCGA database showed that FBXO31 expression is reduced in melanoma tissues compared to normal tissues (Figure 1a). However, CD147 is strongly expressed in melanoma tissues (Figure 1b). The results of CHX tracking experiments showed that the degradation rate of CD147 protein is significantly accelerated in melanoma cells transfected with FBXO31 (Figure 1c). This finding suggests that FBXO31 may regulate the stability of CD147 protein by promoting its degradation. Ubiquitination experiments further demonstrated that FBXO31 increases the ubiquitination level of CD147 (Figure 1d).

### 3.2 FBXO31 is downregulated in A375-SP cells

**Figure 2:**
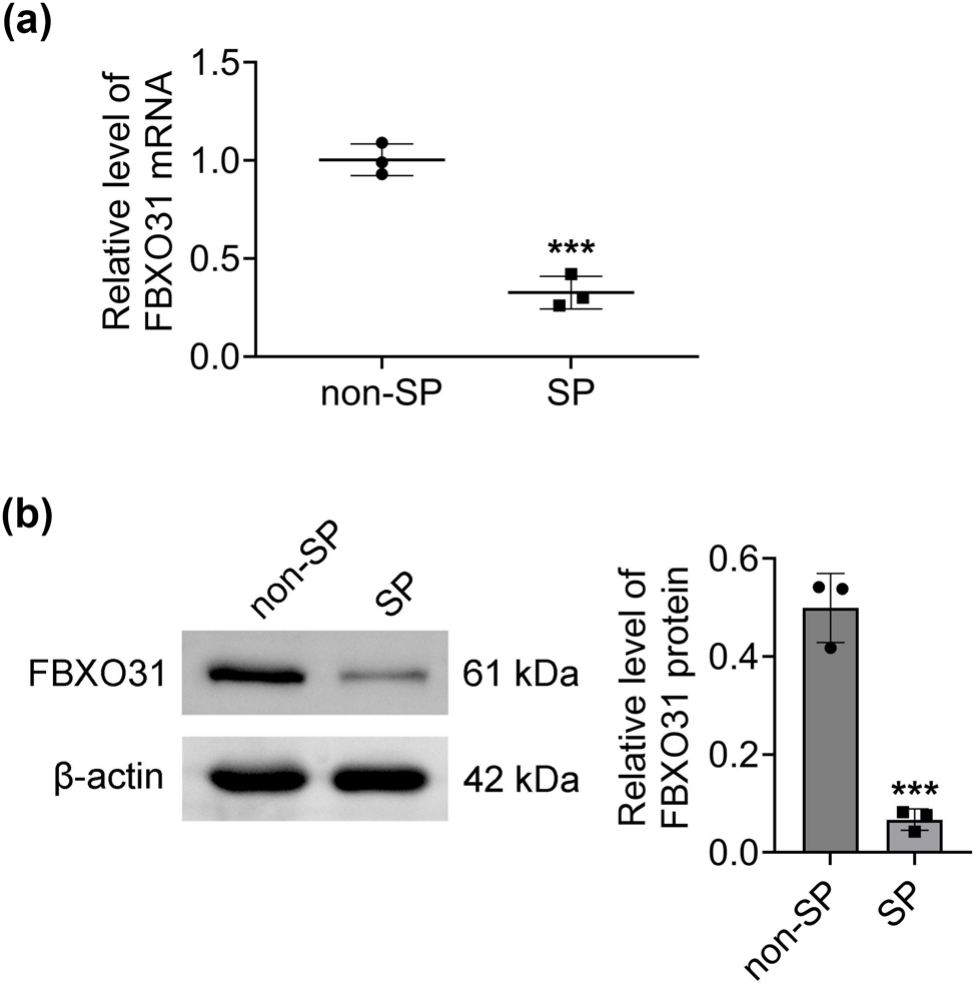
FBXO31 is downregulated in A375-SP cells. (a) Detection of FBXO31 mRNA expression in non-SP cells and SP cells by PCR. (b) Detection of FBXO31 protein expression in non-SP cells and SP cells by Western blot, vs non-SP, ***P < 0.001.

SP cells exhibit tumor stem cell-like properties. PCR and Western blot experiments were used to detect FBXO31 expression in both SP and non-SP cells. The result showed lower levels of FBXO31 mRNA (Figure 2a) and protein (Figure 2b) expression in SP cells.

### 3.3 FBXO31 suppresses the stemness characteristics of CD147 (+) cells

**Figure 3:**
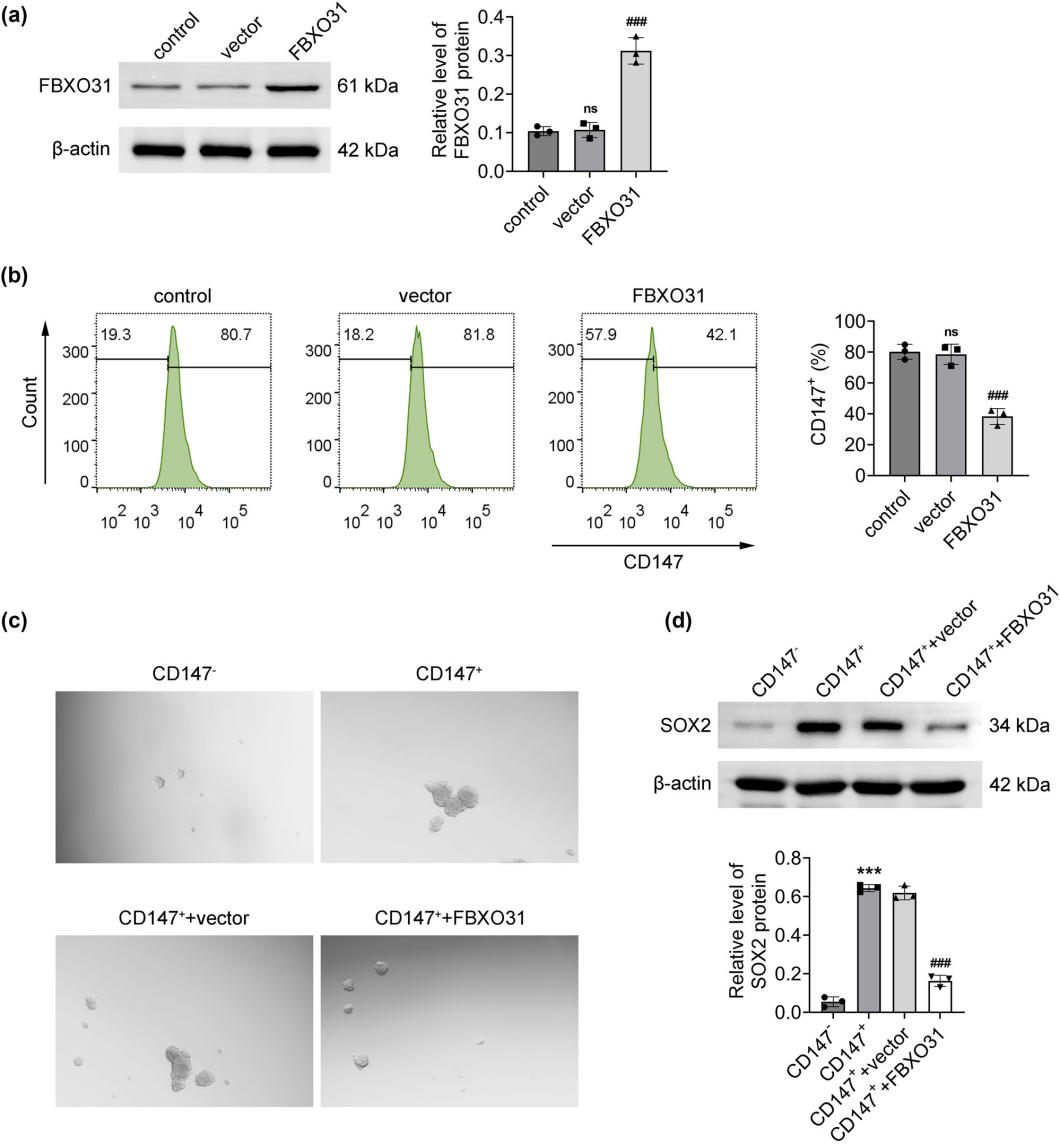
FBXO31 suppresses the stemness characteristics of CD147 (+) cells. (a) Detection of FBXO31 protein expression in SP cells after transfection by Western blot. (b) Flow cytometric sorting of CD147 (+) cells in the SP after FBXO31 transfection. (c) Detection of stemness characteristics of CD147 (+) cells transfected with FBXO31 by sphere formation assay. (d) Western blot was used to detect the protein expression of SOX2 in CD147 (+) cells transfected with FBXO31, vs control, ^###^ *P* < 0.001; vs CD147 (−), ***P < 0.001; vs CD147 (+), ^###^ *P* < 0.001.

After transfection of FBXO31 in SP cells, the transfection effect was detected by Western blot. Successful transfection was indicated by the increased FBXO31 protein expression in cells (Figure 3a). Flowcytometry was subsequently used to sort CD147 (+) cells, which revealed a significant reduction in the proportion of CD147 (+) cells after transfection with FBXO31 compared with the control group (Figure 3b). Following FBXO31 transfection into CD147 (+) cells, sphere formation tests were used to identify the tumor stemness features. SOX2 is a key gene that is essential for maintaining the self-renewal of CSCs. The findings demonstrated that FBXO31 suppressed the capacity of CD147 (+) cells to form spheres (Figure 3c) and express SOX2 (Figure 3d).

### 3.4 FBXO31 inhibits the migration and invasion of CD147 (+) cells

**Figure 4:**
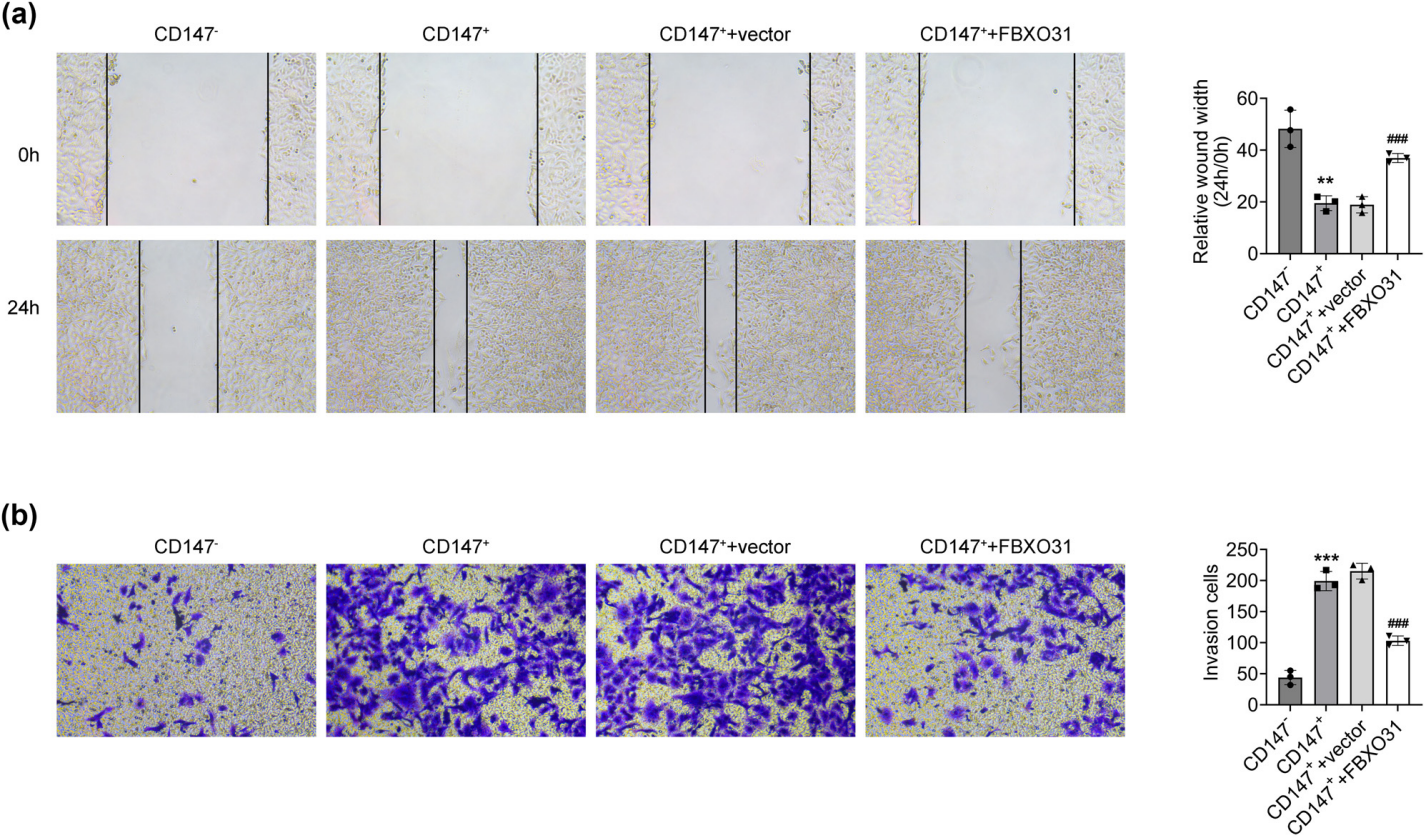
FBXO31 inhibits the migration and invasion of CD147 (+) cells. (a) Wound healing assay to detect the migration ability of CD147 (+) cells transfected with FBXO31. (b) Transwell assay to detect the invasion ability of CD147 (+) cells transfected with FBXO31, vs CD147 (−), **P < 0.01, ***P < 0.001; vs CD147 (+), ^###^ *P* < 0.001.

The scratch (Figure 4a) and Transwell (Figure 4b) assays were used to find the impact of FBXO31 on the migration and invasion capacity of CD147 (+) cells. The findings demonstrated that CD147 (+) cells were more capable of invasion and migration than CD147 (−) cells. Additionally, the invasion and migration capacity of CD147 (+) cells following FBXO31 transfection was diminished.

### 3.5 FBXO31 inhibits the tumorigenicity of CD147 (+) cells *in vivo*

**Figure 5:**
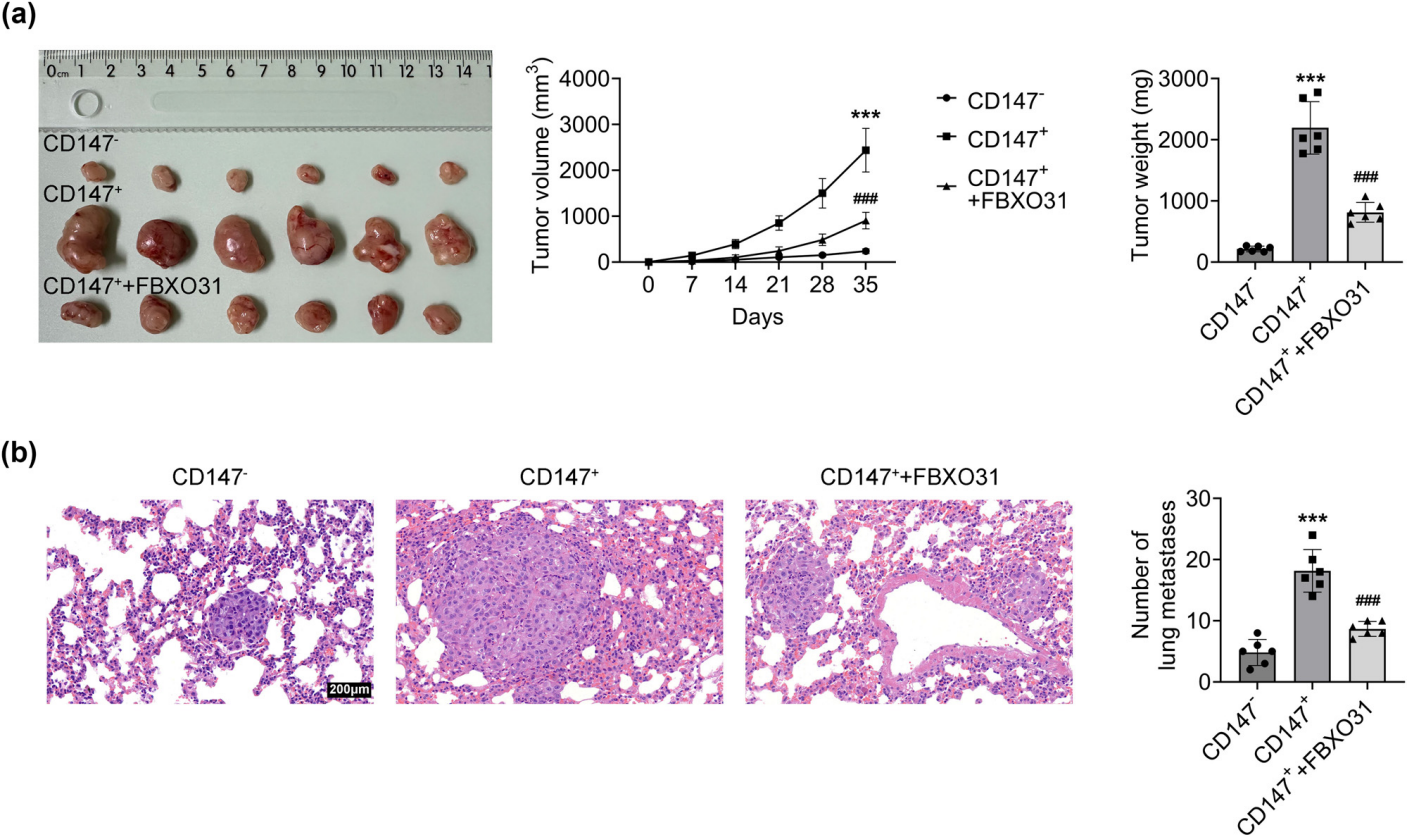
FBXO31 inhibits the tumorigenicity of CD147 (+) cells *in vivo.* (a) Tumor tissue size, volume, and mass of mice in each group. (b) H&E staining to examine lung tissue tumor metastasis, vs CD147 (−), ***P < 0.001; vs CD147 (+), ^###^ *P* < 0.001.

To evaluate the impact of FBXO31 on tumorigenicity, CD147 (−) cells, CD147 (+) cells, and CD147 (+) cells transfected with FBXO31 were subcutaneously injected into nude mice. The tumor growth was monitored over time. The results showed that the tumors of mice injected with CD147 (+) cells became larger with increased volume and mass, while the tumor of mice injected with CD147 (+) and transfected with FBXO31 became smaller with decreased volume and mass (Figure 5a). This shows that FBXO31 can inhibit the tumor growth caused by CD147 (+) cells. In addition, H&E staining (Figure 5b) was used to examine tumor metastasis in lung tissue. Mice injected with CD147 (+) cells exhibited a higher number of metastatic nodules in the lungs, whereas FBXO31 overexpression significantly reduced lung metastases.

## 4 Discussion

CSCs are a subpopulation of tumor cells characterized by their capacity for self-renewal and differentiation, and they have been identified in various cancer types. The development and maintenance of malignancies are significantly influenced by CSCs, often leading to distant metastases and a poor prognosis [13]. Because of this, CSC-targeted therapy holds a promising approach for the treatment of metastatic malignancies.

Several studies have shown that patients with early-stage melanoma exhibit high levels of CD147 expression, which is strongly linked to tumor metastasis and a decreased overall survival rate [14]. Studies have demonstrated that CD147 is critically involved in melanoma progression, promoting angiogenesis, proliferation, and cell migration, while its knockdown suppresses these oncogenic behaviors [15]. Beyond melanoma, research has also indicated that CD147 can be employed as a surface marker to separate breast CSCs. Sorted CD147 (+) cells have been shown to exhibit robust *in vitro* sphere-forming ability, high *in vivo* tumorigenic capacity, robust serum differentiation ability, and increased expression of stemness-related markers like SOX2 [16]. The use of CD147 (+) cells as a marker for CSC sorting has also been validated by earlier findings of our research group. CD147 (+) cells have stronger migration, invasion, and stemness characteristics and can promote tumor growth and metastasis *in vivo*.

The F-box protein FBXO31 is a substrate recognition protein for SCF-class E3 ubiquitin ligases that mediates substrate ubiquitination and degradation. It has been identified as a tumor suppressor gene in both cervical cancer [17] and gastric cancer [18]. Studies suggest that FBXO31 affects the growth of cancer by playing a crucial part in biological processes such as DNA repair, cell cycle, cell growth, and metastasis [19]. Additionally, some research has demonstrated that FBXO31 functions as an oncoprotein that facilitates the development and spread of pancreatic cancer [20].

Despite these findings, the role of FBXO31 in regulating CSC characteristics has remained largely unexplored. In this study, we demonstrate for the first time that FBXO31 expression is significantly downregulated in melanoma SP cells. Moreover, the ability of CD147 (+) cells to form spheres, migrate and invade, and develop and metastasize *in vivo* was significantly diminished due to overexpression of FBXO31. All these findings collectively suggest that FBXO31 attenuates the stemness features of CD147 (+) melanoma stem cells.

Despite the promising findings, this study has certain limitations. Clinical skin melanoma tissue samples were not collected to validate the expression levels of FBXO31 and CD147. Future research will aim to address this by testing each of these individually in clinical samples. Additionally, CSCs are closely associated with glycolysis and tumor resistance. Further investigations are warranted to explore the relationship between FBXO31, tumor metabolism, and drug resistance in melanoma.
